# Comprehensive androgen-dependent transcriptome analysis in human genital tissue

**DOI:** 10.1186/s12864-025-12212-6

**Published:** 2025-11-17

**Authors:** Radhika Sivaprasad, Kristian Händler, Almuth Caliebe, Malte Spielmann, Paul-Martin Holterhus, Nadine C. Hornig

**Affiliations:** 1 Institute of Human Genetics, University Hospital of Schleswig-Holstein, Kiel University, Kiel, Germany; 2 Institute of Human Genetics, University Hospital of Schleswig-Holstein, University of Lübeck and Kiel University, Lübeck and Kiel , Germany; 3 Department of Paediatric Oncology and Rheumatology, Paediatric Endocrinology and Diabetes, University Hospital of Schleswig-Holstein, Kiel, Germany

**Keywords:** Androgen receptor, Androgen insensitivity syndrome, Transcriptional regulation

## Abstract

**Background:**

Androgen signalling through the androgen receptor (AR) is crucial for male genital development. Disruptions in this pathway are associated with androgen insensitivity syndrome (AIS), which is typically caused by mutations in the AR gene, although the underlying genetic mechanisms remain unknown in many cases. To better understand androgen-dependent transcriptional changes in human genital tissue, we performed transcriptomic profiling of foreskin- and scrotum-derived human genital skin fibroblasts (GSFs) treated with dihydrotestosterone.

**Results:**

Differential gene expression analysis revealed 409 and 260 reproducibly up-regulated genes in foreskin- and scrotum-derived GSFs, respectively. GSFs from individuals with complete androgen insensitivity syndrome, carrying inactivating mutations in the *AR* gene, showed no reproducible androgen response. Androgen response element motif scanning confirmed direct AR binding in key up-regulated genes, including *AOX1*, *APOD*, *FKBP5*, and *FAM107A*. Gene ontology analysis revealed enrichment in pathways related to neuronal, muscle, cardiovascular, and sex development.

**Conclusion:**

Identifying new AR target genes broadens the current understanding of androgen signalling and aids in better understanding the aetiology of AIS, and other androgen-related conditions.

**Supplementary Information:**

The online version contains supplementary material available at 10.1186/s12864-025-12212-6.

## Background

Androgens play an essential role in the development and functional maintenance of male reproductive tissues. Their physiological effects are mediated through the androgen receptor (AR), a 110-kDa protein belonging to the nuclear receptor family of ligand-activated transcription factors [1]. Upon binding of testosterone (T) or the more potent androgen dihydrotestosterone (DHT), cytoplasmic AR translocates into the nucleus and binds to androgen-responsive DNA elements (AREs), thereby regulating the transcription of androgen-dependent genes. Changes in this pathway are associated with androgen insensitivity syndrome (AIS) [2, 3].

AIS is an X-linked recessive condition and a common cause of 46,XY differences of sex development (DSD). The phenotype varies from female external genitalia in the complete form (CAIS) to male external genitalia with infertility and/or gynecomastia in the mild form (MAIS). Partial androgen insensitivity syndrome (PAIS) exhibits a broad spectrum of undervirilized male external genitalia ranging from isolated micropenis to severe hypospadias [2].

AIS is classically caused by disruptive mutations in the *AR* gene and the vast majority of CAIS individuals carry hemizygous mutations in the *AR*. However, an *AR* mutation can be found in less than 40% of individuals diagnosed with PAIS, suggesting that factors outside the *AR* may contribute to a similar phenotype [4]. Measuring DHT-induced expression of the endogenous AR-target gene apolipoprotein D (*APOD*) in genital skin fibroblasts (GSFs) from individuals with the suspected diagnosis AIS but no mutation in the *AR* gene revealed a group of GSFs with reduced AR activity, termed AIS type II [5]. This prompted the hypothesis that co-factors of the AR when mutated could lead to an AIS-like phenotype. The transcriptional activity of the AR is influenced by many cofactors that can exert their regulatory role by direct interaction with the AR or through post-translational modifications like phosphorylation or ubiquitination [6–8]. Mutations in AR interacting proteins have been identified in prostate cancer, but the composition of the AR transcriptional complex required for masculinization during human embryogenesis is largely unknown. In vitro studies identified RWDD1 as direct transcriptional activator of the AR [9]. Interestingly, RWD domains containing proteins have been implicated in ubiquitin- and SUMO-dependent processes and it has been speculated that RWDD1 functions as AR coactivator by participating in a ubiquitin- or SUMO-mediated process [9]. Recently, the first genetic cause of AIS type II was described, identifying the formin DAAM2 as a cofactor of the AR, necessary for its transcriptional activity [10]. Another possible cause of AIS type II could be changes in transcriptional targets of the AR. Previous AR-dependent gene expression analyses in GSF from male controls and CAIS individuals revealed androgen-dependent programming in these cells [11]. Inactivating mutations of the AR appear to induce lasting changes in gene expression in cultured fibroblasts as well as peripheral blood mononuclear cells (PBMC) indicating long-term sex-specific hormonal programming [12, 13]. Androgen stimulation by hCG treatment in young boys showed an increase in several non-coding small RNAs in PBMCs [14]. Subsequent microarray-based studies aimed at identifying DHT-induced AR target genes in GSFs, revealed very few significantly up-regulated genes [15, 16] one of which, *APOD*, has been validated in a large cohort [5]. Using optimized endogenous AR-activity assay conditions, we performed mRNA sequencing on untreated and DHT-treated GSFs derived from six foreskin biopsies and six scrotum biopsies of male controls. We also included GSFs from four CAIS individuals, who lack AR activity, serving as ideal controls to identify gene expression changes mediated by the AR. We show a comprehensive analysis of androgen-dependent transcriptional changes in human genital tissue, identify AR-target genes that are either tissue-specific or common to both tissues and discuss how these transcriptional changes can affect androgen-induced pathways.

## Results

### Tissue-specific and hormone-responsive gene expression patterns in GSFs

To identify AR target genes in GSFs, we treated 12 male control cells (six from foreskin and six from scrotum) for 48 h and 72 h with either DHT or vehicle (EtOH). As an *AR*-negative control, we included GSFs from four individuals with CAIS carrying loss-of-function mutations in the *AR* (Supplementary file 1: Fig. S1 and Table S1). All samples were processed for mRNA sequencing and subsequent data analysis.

**Fig. 1:**
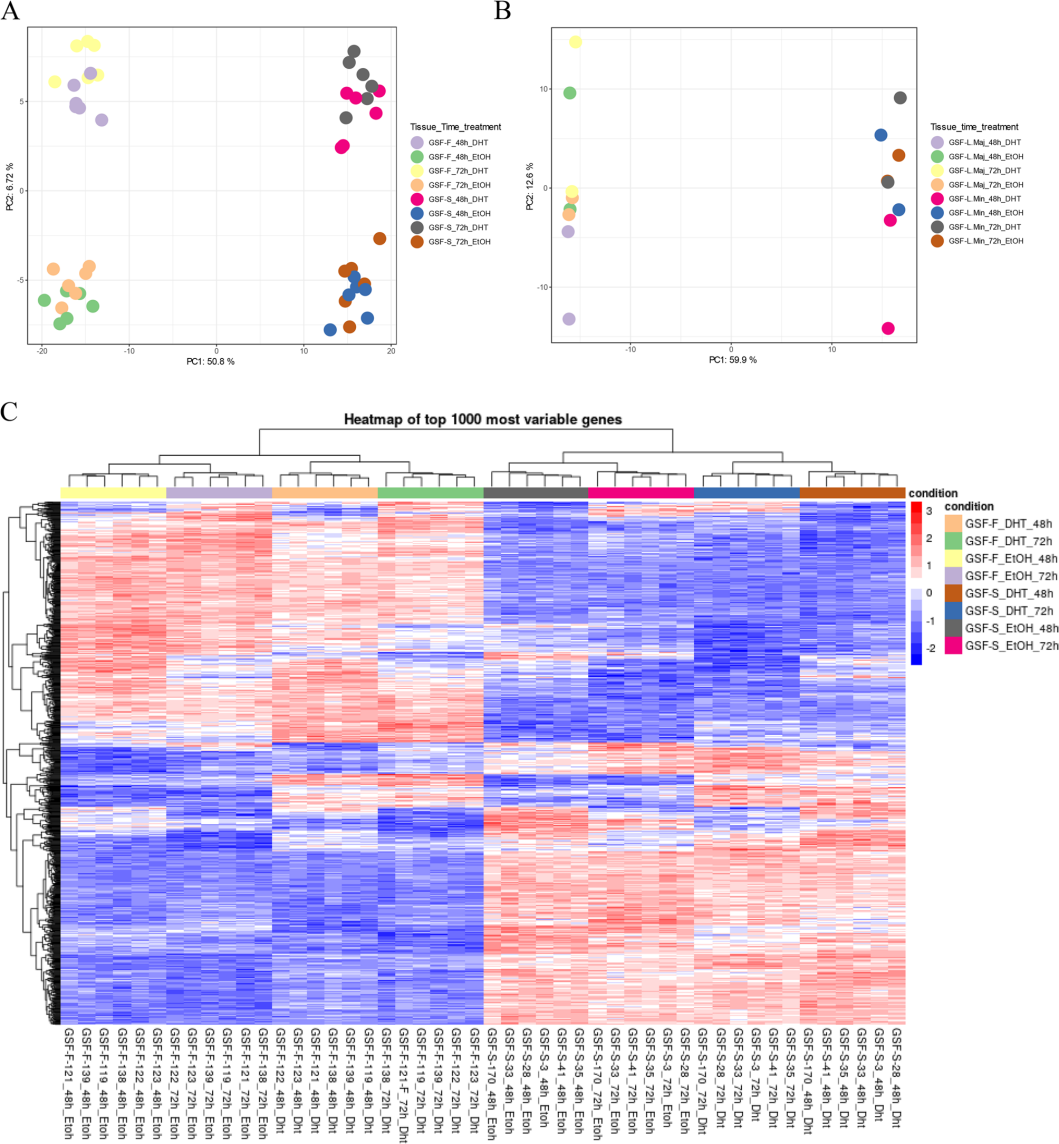
Expression profile of DEGs.** A)**Principal Component Analysis (PCA) of batch-corrected male control samples, showing segregation based on tissue type and treatment. **B)** PCA of batch-corrected CAIS samples, showing segregation based on tissue type. In both PCA plots, each dot represents a single individual (n = 6), color-coded by treatment (EtOH or DHT) and time point (48 h or 72 h). **C)** Heatmap displaying the expression profiles of the top 1,000 most variable genes across all conditions in male control samples. Each column represents an individual sample, grouped by tissue type (foreskin or scrotum), treatment (DHT or EtOH), and time point (48 h or 72 h), as indicated by the color-coded bar above. Each row corresponds to a gene. The colour scale reflects gene expression levels, with red representing high expression and blue indicating low expression. Hierarchical clustering is shown for both genes and samples to visualize expression similarities and condition-specific patterns

Principal component analysis (PCA) of male control samples (Fig. 1A) revealed a primary separation by tissue type (scrotum vs. foreskin), accounting for 50.8% of the variation, and, to a lesser extent, by treatment (DHT vs. EtOH), contributing 6.72% of variation. In CAIS samples (Fig.1B), separation was observed by tissue type (Labia Majora vs. Labia Minora), representing 59.9% of the variation.

To gain a more detailed picture of gene expression variation, we visualized gene expression differences in a heatmap. As shown in Fig. 1C, consistent with the PCA results, the most prominent separation in gene expression in male control samples was based on the tissue type. Within each tissue type, additional differences emerged based on treatment, with some regions showing higher expression in DHT-treated samples compared to EtOH-treated, and vice versa. Furthermore, the duration of treatment (48 h and 72 h) contributed to subtle shifts in expression patterns. In CAIS samples, gene expression was primarily separated by tissue type, with minor changes observed in expression patterns over time (Supplementary file 1: Fig. S2).

### Comparative gene expression analysis

**Fig. 2:**
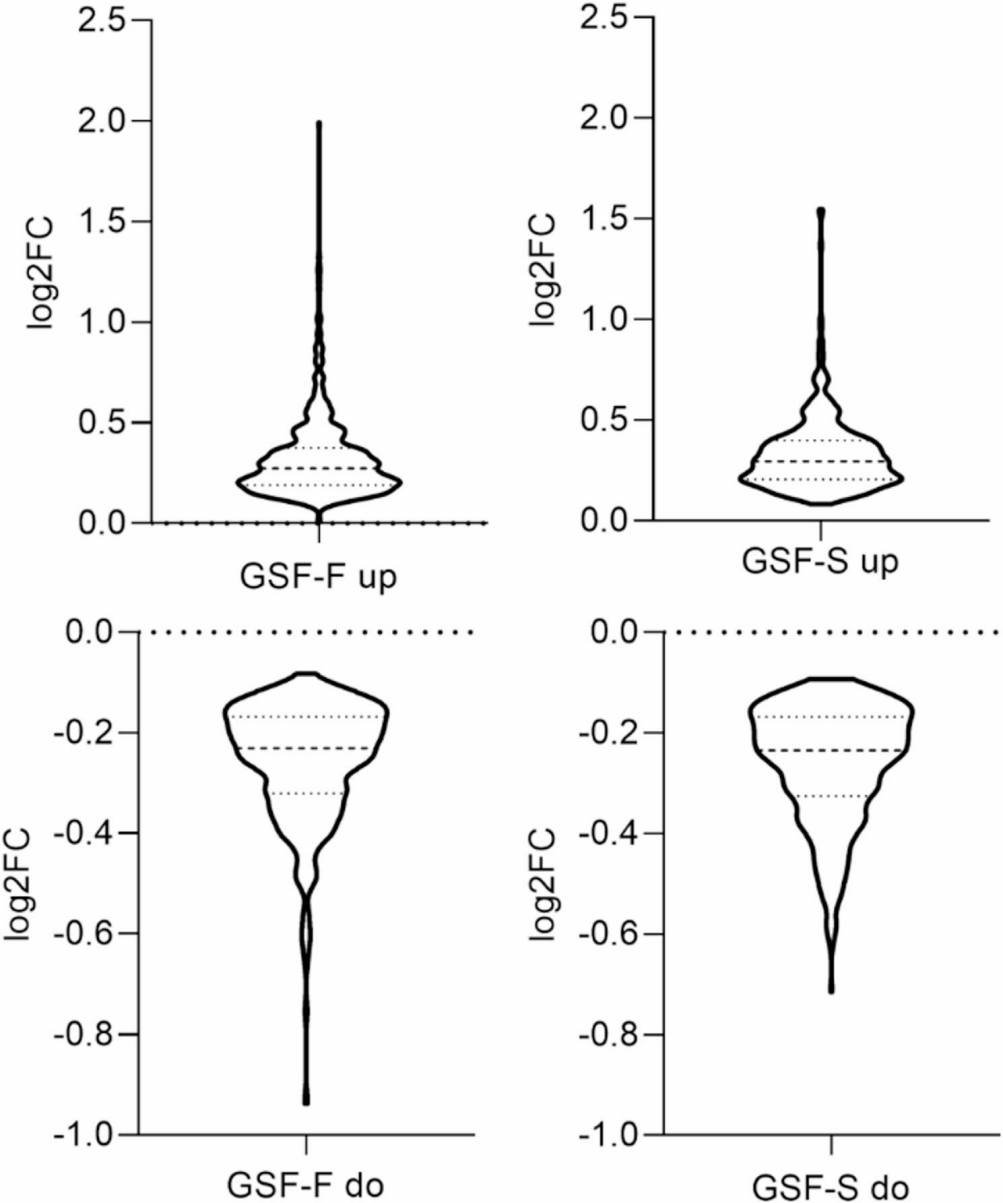
Distribution of DEGs in male control samples based on Log2FC and padj ≤ 0.05. Medians and Quartiles are shown as dotted lines

To investigate the biological basis of the observed clustering, differential gene expression analysis was performed by including all genes with a log2FC above or below zero (padj ≤ 0.05). Foreskin derived samples (GSF-F) displayed the most robust response, with 576 genes up-regulated and 565 down-regulated at 48 h, increasing slightly at 72 h to 602 up-regulated and 576 down-regulated genes. Scrotum-derived samples (GSF-S) also showed substantial changes, with 387 genes up-regulated and 400 down-regulated at 48 h, and this number increased at 72 h to 399 up-regulated and 495 down-regulated genes. In total, this resulted in 1141 and 787 differentially expressed genes (DEGs) at 48 h, and 1178 and 894 genes at 72 h, in GSF-F and GSF-S, respectively (Table 1). The majority of changes were small, with a median log2FC of 0.27 for GSF-F and 0.21 for GSF-S for up-regulated genes and a median log2FC of −0.23 for GSF-F and GSF-S for down-regulated genes (Fig. 2).

In CAIS, three differentially expressed genes were identified in labia minora derived samples and five in labia majora derived samples at 72 h, while none were identified at 48 h (Table 1). The transcripts did not overlap with any differentially expressed genes in the male control cohort. The observation that nearly no androgen-dependent gene expression changes were observed in CAIS samples indicates that the transcriptional changes observed in male control samples were indeed AR-dependent.

**Table 1:** Summary of DEGs in DHT- versus EtOH-treated GSFs across tissue types and time points

Sample Condition (DHT vs. EtOH)	No. of. up-regulated genes	No. of. down-regulated genes	Total
GSF-F_48 h	576	565	1141
GSF-F_72 h	602	576	1178
GSF-S_48 h	387	400	787
GSF-S_72 h	399	495	894
GSF-L.Min_48 h	0	0	0
GSF- L.Min _72 h	1	2	3
GSF-L.Maj_48 h	0	0	0
GSF- L.Maj _72 h	2	3	5

**Fig. 3:**
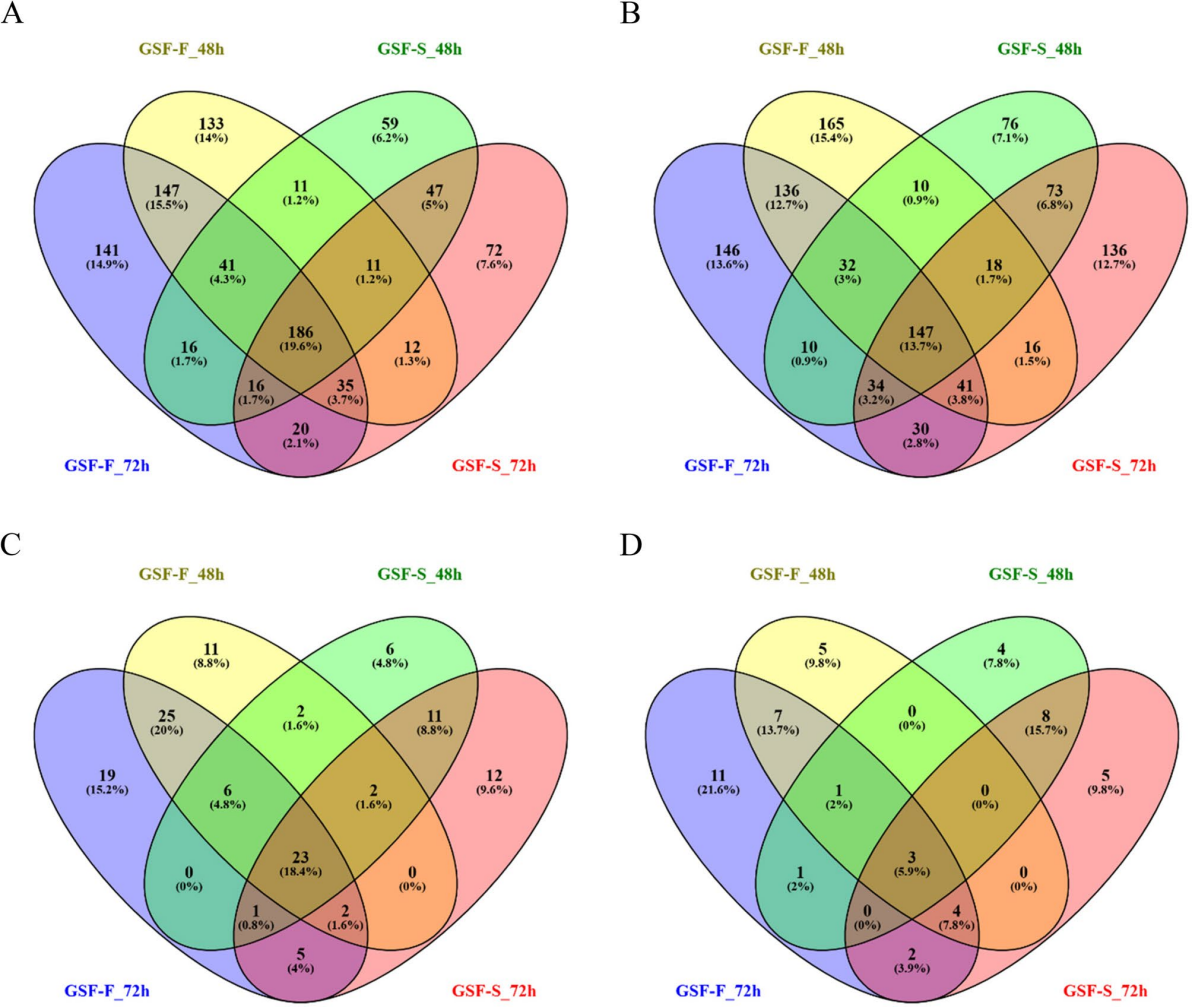
Venn diagram illustrating the individual numbers, percentages and overlaps of **A)** up-regulated and **B)** down-regulated genes without any threshold, padj ≤ 0.05; **C)** up-regulated and **D)** down-regulated genes with a threshold log2FC ≥ 0.5 or log2FC ≤−0.5 and padj ≤ 0.05 in foreskin and scrotum derived GSFs following 48 and 72 hours of DHT treatment

In GSF-F 409 genes (53.2%) were commonly up-regulated and 356 genes (45.4%) were commonly down-regulated at both 48 h and 72 h of treatment (Fig. 3A, B and Supplementary file 1: Fig. S3A, C). In GSF-S 260 genes (49.4%) were commonly up-regulated and 272 genes (43.7%) were commonly down-regulated (Fig. 5A, B and Supplementary file 1: Fig. S3B, D) at both time points. A total of 186 (19.6%) of genes were up-regulated at both timepoints in both tissues, while 147 genes (13.7%) were down-regulated (Fig. 3A, B).

**Fig. 4:**
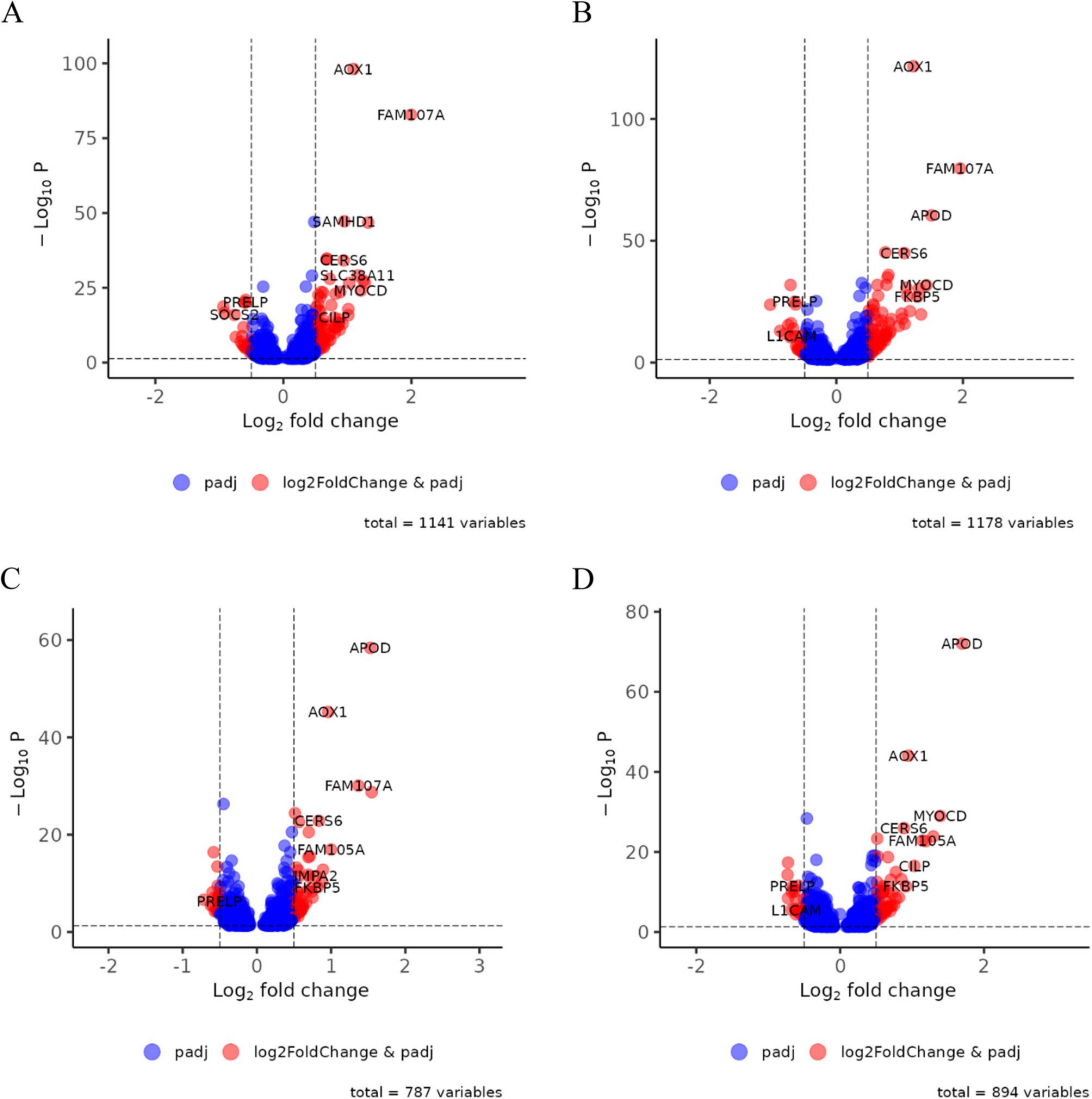
Volcano plots displaying the distribution of DEGs in **A)** GSF-F_48 h, **B)** GSF-F_72 h, **C)** GSF-S_48 h, and **D)**GSF-S_72 h, comparing DHT to EtOH treatment. In all plots, red dots represent significant DEGs that meet both the log2FC ≥ 0.5 or log2FC ≤ −0.5 and padj ≤ 0.05 thresholds. Blue dots represent genes that meet only the significance threshold but not the log2FC threshold. Dotted lines indicate the log2FC threshold of ±0.5

For a more stringent analysis, only genes with a log2FC ≥ 0.5 or log2FC ≤ −0.5 and padj ≤ 0.05 were considered. This approach reduces false positives as well as indirect effects, allowing a focus on the most robustly regulated transcripts. In GSF-F, 56 genes (58.3%) were commonly up-regulated and 15 genes (44.1%) were commonly down-regulated (Fig. 3C, D and Supplementary file 1: Fig. S4A, C). In GSF-S, 37 genes (52.9%) were commonly up-regulated and 11 genes (39.3%) commonly down-regulated (Fig. 3C, D and Supplementary file 1: Fig. S4B, D). Across all conditions, 23 genes (18.4%) were commonly up-regulated and 3 genes (5.9%) were commonly down-regulated (Fig. 3C, D). No DEGs with a log2FC ≥ 0.5 or log2FC ≤ −0.5 and padj ≤ 0.05 were observed in CAIS derived samples. The list of DEGs is provided in Supplementary file 2.

Several genes showed a strong and consistent regulation in all conditions. Among the most significantly up-regulated genes were *AOX1*, *FAM107A*, *CERS6* and *APOD* which appeared in multiple comparisons (Fig. 4A-D). Other recurrently up-regulated genes included *FAM105A*, *MYOCD* and *FKBP5*. Conversely, *PRELP*, and *L1CAM* appeared repeatedly among the down-regulated genes across various conditions and tissues (Fig. 4A-D). We validated the DHT-induced changes in gene expression for six up-regulated genes (*STEAP4*, *MYOCD*, *FAM105A*, *FAM107A*, *HSD11B1*, *FKBP5*) by quantitative PCR (qPCR) (Supplementary file 1: Fig. S5 and Supplementary file 3).

### AR-chromatin binding and binding motif scan within DEG regions

In order to verify if the commonly up- and down-regulated genes are directly targeted by the AR, we screened publicly available AR chromatin immunoprecipitation sequencing (ChIPseq) data [17, 18] for DHT-dependent AR-binding in the prostate cancer cell lines LnCaP and VCaP. Although these cells derive from a different tissue, we expected to see some overlap in AR-dependent transcription using the most robust AR target genes found in our analysis (those commonly up- or down-regulated at both time points in both tissues with a minimum log2FC ≥ 0.5 or ≤ −0.5). Of the 23 up-regulated genes, 12 (52%) showed AR chromatin binding according to the ChIPseq data. No down-regulated gene showed AR chromatin binding. The majority of binding sites were intragenic with a few sites upstream the transcriptional start site (Table 2). We validated the binding site by scanning sites for the canonical AR binding site motif (AGAACANNNTGTTCT). At all sites high scoring motifs were found (*p* < 0.001). The complete list of genes and their associated AR binding sites is provided in Supplementary file 1: Table S2.

**Table 2:** AR binding near DEGs in GSF-F/S with canonical AREs. TSS = transcriptional start site

Up-regulated genes (GSF-F and GSF-S at 48 h and 72 h)	AR ChIPseq peak in LnCaP (distance to TSS in bp)	AR ChIPseq peak in VCaP (distance to TSS in bp)	Canonical AR binding site motif: AGAACANNNTGTTCT	*p*-value/q-value
*AOX1*	+ 80,015	+ 80,015	AGAACAATCTGTTAG	4.01e-05/0.0104
*APOD*	−485	−485	GGAACATGGAGTTCC	8.62e-05/0.0464
*CCDC68*	+ 1927	+ 1927	AGAACACAGTGTCCT	8.35e-07/0.000231
*CD82*	−932	−932	AGCACTGGTTGTTCT	9.95e-06/0.0232
*CERS6*	+ 71,232	+ 71,232	AGAACACTCTGTGCT	8.35e-07/0.001
*ERCC6*	+ 34,037	+ 34,037	AGAGCATGCTGTTTT	2.69e-05/0.0248
*FAM105A*	+ 7808	+ 7808	AGGACACCGTGTGCT	4.49e-06/0.00344
*FAM107A*	+ 205	+ 205	GGAACATCATGTCCA	0.000142/0.046
*FKBP5*	+ 1682	+ 1682	GGAACACGAGGTTCT	9.95e-06/0.00476
*IMPA2*	−4365, −4931	−4365, −4931	AGAAAAAGCTGATTT, TGGCCAGGCTGGTCT	0.000376/0.0899, 0.000524/0.089
*KIF26B*	+ 24,860	+ 24,860	AGAACATCCTGTCCA	9.95e-06/0.00813
*MYOCD*	+ 15,790	+ 15,790	AGAACAGTGTGTACC	9.95e-06/0.00889

### Functional categorization and pathway enrichment of DEGs

**Fig. 5:**
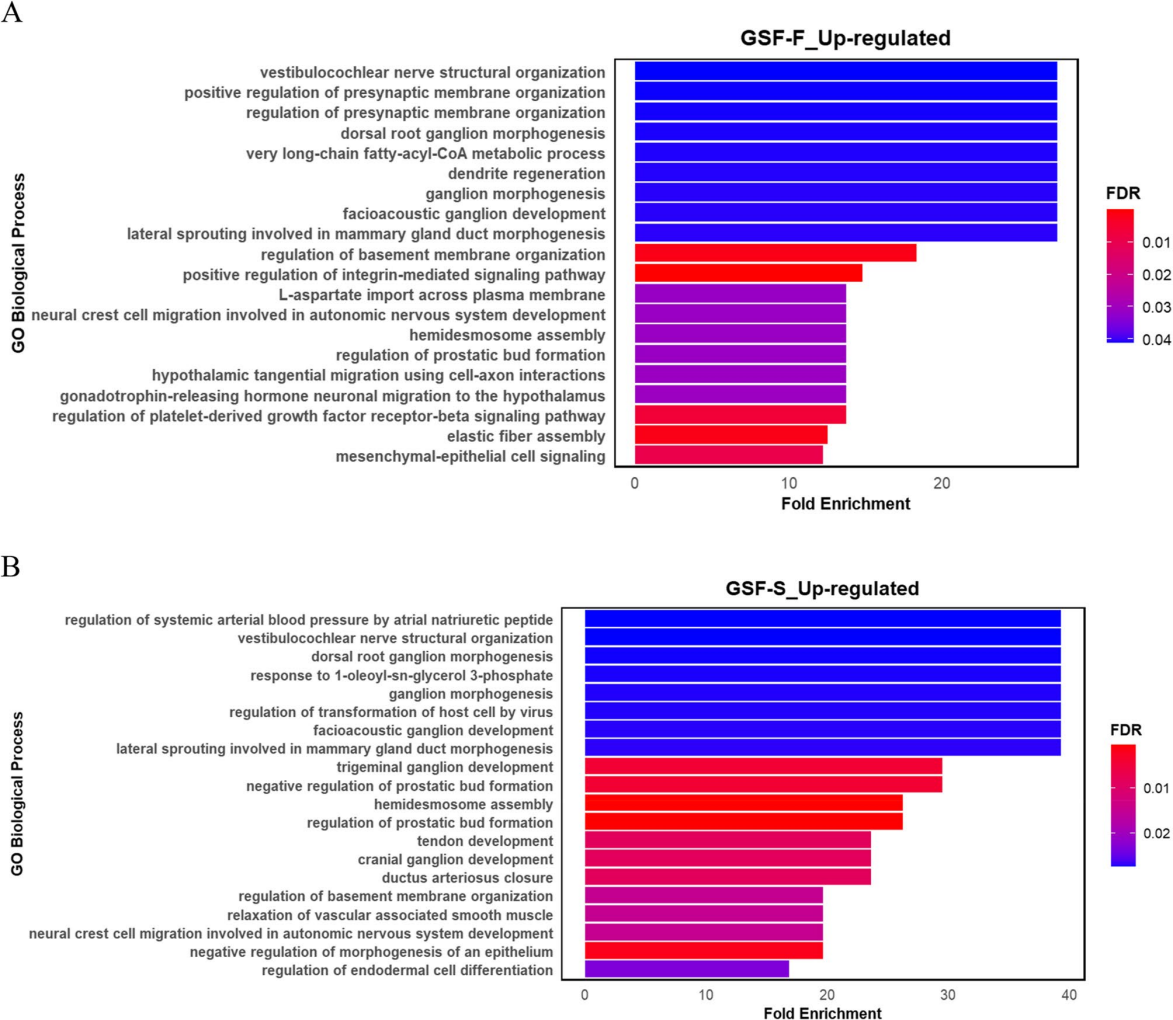
Bar plot depicting top enriched Gene Ontology biological process terms based on fold enrichment for up-regulated DEGs in **A)** GSF-F and **B)** GSF-S. Bar lengths indicate fold enrichment, and colour gradients represent FDR values, with darker red indicating higher statistical significance and blue indicating lower statistical significance

Gene Ontology Analysis (GOA) of all up-regulated genes (FDR < 0.05) revealed enrichment in several processes related to neurodevelopment, such as ganglion morphogenesis, across both foreskin- and scrotum-derived samples. Additionally, GO processes such as hemidesmosome assembly, lateral sprouting involved in mammary gland duct morphogenesis, regulation of prostatic bud formation and regulation of basement membrane organization were shared between the two groups (Fig. 5A, B). In contrast, distinct sets of GO terms were enriched in each tissue. In GSF-F (Fig. 5A), processes such as regulation of presynaptic membrane organization and gonadotrophin-releasing hormone neuronal migration to the hypothalamus were enriched. Meanwhile, GSF-S (Fig. 5B) exhibited enrichment for processes such as relaxation of vascular associated smooth muscle and regulation of systemic arterial blood pressure.

**Fig. 6:**
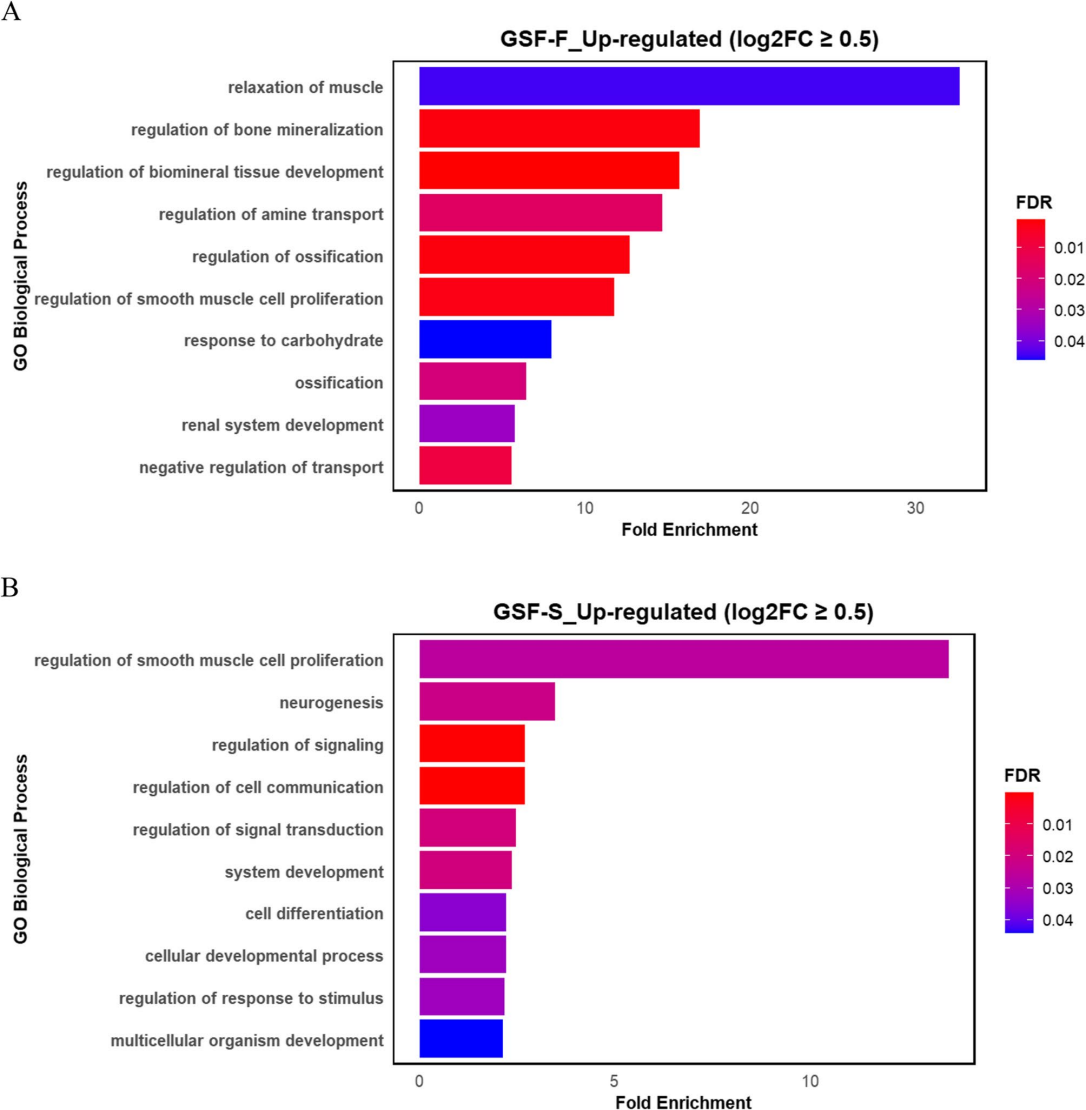
Bar plot depicting the top enriched Gene Ontology biological process terms based on fold enrichment for up-regulated DEGs with the log2FC≥ 0.5 in **A)** GSF-F and **B)** GSF-S. Bar lengths indicate fold enrichment, and colour gradients represent FDR values, with darker red indicating higher statistical significance and blue indicating lower statistical significance

We also performed GOA on up-regulated genes with a log2FC of ≥ 0.5. In GSF-F biological processes related to bone tissue development or remodelling were significantly enriched (Fig. 6A). Whereas, in GSF-S, signalling and cell communication were a prominent theme (Fig. 6B). The list of all GO Biological Process terms is provided in Supplementary file 4.

## Discussion

Androgens are essential for male genital differentiation, as demonstrated by individuals with androgen insensitivity syndrome, who often exhibit reduced or absent genital virilization at birth due to reduced or absent AR activity. While androgen-activated gene transcription drives key physiological changes during male embryonic development, these gene programs have not yet been fully understood in humans. In mice, androgen-dependent gene expression during male genital development has been studied by comparing male and female cell populations in the external genitalia during the critical sex differentiation window in the mouse embryo [19]. Given that for ethical reasons such experiments are not possible in humans, GSFs, deriving from an androgen sensitive tissue, represent a valuable research material. Although being terminally differentiated they still might reflect some of the developmental processes occurring during the critical time window where androgens act to induce male sex differentiation. Large-scale AR-dependent gene expression programs in GSF from male controls and CAIS individuals revealed distinct transcriptional profiles, suggesting androgen-dependent programming in these cells [11]. Two further studies used GSFs to identify androgen-induced target genes through microarray analysis with the identification of *APOD* as a *bona fide* AR target. We here used mRNA sequencing to identify additional DHT-induced genes in both foreskin- and scrotum-derived GSFs and compared them to GSFs derived from CAIS individuals.

Our study revealed a tissue specific response to DHT treatment, with foreskin-derived GSFs showing a higher number of differentially expressed genes compared to scrotum-derived GSFs. The majority of androgen-induced changes are, although being significant, subtle and might be secondary changes. Considering only changes with log2FC ≥ 0.5 or ≤ −0.5, foreskin derived fibroblasts showed 56 consistently up-regulated and 15 down-regulated genes, while in scrotal skin derived fibroblasts the numbers of DEGs were 37 and 11, respectively. Across all conditions, 23 genes were commonly up-regulated and 3 genes were commonly down-regulated, making them very robust AR-targets. No reproducible DHT-dependent transcriptional changes were observed in samples from CAIS individuals supporting the notion that the transcriptional changes identified in male control samples are indeed AR-dependent.

Among the 23 commonly androgen-induced genes, several have previously been linked to androgen signalling. *APOD* is androgen-responsive in GSF, though its role in male genital development remains unclear [15]. It is a glycoprotein mainly associated with high-density lipoproteins in human plasma and belongs to the lipocalin family, which transport small hydrophobic molecules. It can bind cholesterol, progesterone, pregnenolone, bilirubin, and arachidonic acid, though its physiological ligands remain uncertain [20]. It also plays a role in oxidative stress, inflammation, and lipid metabolism [21, 22]. The *APOD* gene is broadly expressed, with high levels in the adrenal glands, pancreas, kidneys, placenta, spleen, lungs, reproductive organs and the brain [20]. Interestingly, APOD is also present in apocrine axillary secretions. Human studies have proposed that axillary odours and secretions from both sexes may serve as sources of chemical signals within and between sexes [23].

FKBP5 is part of the HSP90 chaperone complex, a complex that keeps un-ligated AR in the cytoplasm [24]. Depletion of either *FKBP5* or *FKBP4* in prostate cancer cells reduces AR dimer formation, chromatin binding, and phosphorylation, suggesting defective AR signalling [25]. The AR, in turn, directly regulates the *FKBP5* gene via a distal enhancer element indicating a regulatory feedback mechanism [26]. FKBP5 is therefore both a cofactor of the AR and a direct target gene of it through which AR activity is regulated. In the presence of high androgen levels, more FKBP5 is needed for proper AR protein folding which is ensured through the androgen-induced upregulation of *FKBP5*. Reduced *FKBP5* expression or activity could therefore lead to reduced AR activity, as seen in AIS. Monoamine oxidase A (MAOA) plays a significant role in prostate cancer progression and AR signalling. MAOA and AR form a positive feedback loop, with androgens inducing *MAOA* expression through AR binding to the *MAOA* gene, while MAOA enhances AR transcriptional activity [27]. A link to male sex development has not been shown so far.

FAM105A is an inactive pseudodeubiquitinase of the ovarian tumour protease (OTU domain) family in humans and is thought to function through its ability to mediate protein-protein interactions [28]. Ubiquitinases have been shown to tightly interact with chaperones, targeting unfolded proteins for degradation, and AR protein ubiquitination happens within the chaperone complex [8]. FAM105A protein might therefore play a role in chaperon-mediated AR folding and turn-over but so far evidence is missing. *FAM107A*, which is functionally unrelated to *FAM105A* has previously been found to be DHT-regulated in foreskin derived fibroblasts [16] as well as neural stem cells [29]. FAM107A is a stress-inducible actin-binding nuclear protein thought to act as a transcriptional regulator and has been shown to interact with the histone acetyltransferase complex [30]. As an acting binding protein, it was suspected to have a role in cytoskeleton organization although this does not align with its nuclear localisation. The role of nuclear actin in AR-mediated transcription has been shown before [10] with the formin DAAM2 being a co-factor of AR-mediated transcription. It could therefore be that FAM107 exerts its actin binding capacity in the nucleus, but the role of FAM107 in AR signalling remains to be elucidated.

Aldehyde Oxidase 1 (AOX1) is a cytosolic enzyme highly expressed in liver playing a key role in metabolizing drugs. The human protein atlas also shows an enrichment of AOX1 in male tissues. Evidence from immature mice and from castrated and testosterone-treated animals supports the role of testosterone in increasing N1-methylnicotinamide oxidation [31]. This oxidation has later been shown to be catalysed by Aldehyde Oxidase [32]. The increase in oxidase activity which normally occurs in maturing males can be initiated prematurely, delayed, reversed, or prevented by experimental manipulation of testosterone availability [31, 33]. Similarly, we see an increase in *AOX1* expression upon DHT treatment in genital skin fibroblasts. Nevertheless, the role of androgens in inducing aldehyde oxidase activity and drug metabolism is still unclear.

Considering all up-regulated genes with a log2 fold change equal or above 0.5 in both tissues, *SLC12A2*, *ACTG2*, *HGF*, and *HAND2* have been associated with male genital development.

Male *Slc12a2*-deficient mice are infertile due to defective spermatogenesis and *Slc12a2* is expressed in Sertoli cells in wild-type animals [34]. Mutations in *ACTG2* are associated with genitourinary phenotypes like hydroureter, megacystis and undescended testes (OMIM #619431). Hepatocyte growth factor (HGF) is a key regulator of organ development and homeostasis, with growing evidence of its role in testicular physiology before and after birth. The HGF system is active throughout all stages of testis development, influencing processes such as steroidogenesis, apoptosis, mitosis, morphogenesis, and differentiation. Overall, HGF appears to be an important factor in development and the regulation of spermatogenesis [35].

HAND2 is a basic helix–loop–helix transcription factor essential for embryonic development. It plays key roles in cardiac morphogenesis, as well as in limb and craniofacial patterning through regulation of Sonic hedgehog (SHH) signalling [36]. SHH is an important morphogen in external genital formation. It is expressed in the urethral plate epithelium of the genital tubercle, where it regulates outgrowth and patterning. Mice with a targeted deletion of SHH, lack external genitalia development [37, 38]. Of note, in non-mammalian vertebrates, androgens regulate HAND2, which drives male clasper (copulatory organs) development via SHH signalling [39]. In summary, there is evidence that some of the here described DHT-induced genes might have a direct role in male sexual differentiation. The identification of variants in these genes in individuals with 46,XY DSD and suspected diagnosis of AIS is the next step to validate their role in male sex development.

Gene ontology analysis gives a broader picture about androgen induced biological pathways. In both tissue types GO terms related to sex differentiation are present, in GFS-F also the term “response to steroid hormone”, although these terms are not under the most enriched ones. Under the twenty most enriched terms in both GSF-F and GSF-S is “regulation of prostatic bud formation”. Prostatic buds originate from the urogenital sinus epithelium in the developing foetus. Androgens play a critical role in promoting prostatic bud development, including their elongation and branching [40].

In foreskin derived GSF, the most enriched GO terms converge on coordinated cellular processes for the development, organization, and regulation of complex tissues, especially regarding the nervous system, followed by the cardiovascular/vascular system. Of particular interest is the GO term “gonadotrophin-releasing hormone neuronal migration to the hypothalamus”, as gonadotrophin-releasing hormone from the hypothalamus activates gonadal steroid hormone synthesis and is negatively regulated through androgen signalling [41]. A disrupted hypothalamus-gonadal axis can be a cause of DSD by impairing the development and function of the gonads. GO-terms derived from the more stringent analysis of genes up-regulated ≥ 0.5 log2FC relate to the formation and regulation of organs and tissues, particularly the skeletal (e.g., ossification and bone mineralization), followed by muscle, renal and nervous systems. Further enriched terms relate to cell signalling and cell motility. In scrotum-derived GSF, the most enriched GO terms share common themes centred on the development, structural organization, and functional regulation particularly within the nervous, cardiovascular, and muscular systems overlapping partly with androgen induced biological processes found in GSF-F.

In summary, although being terminally differentiated, androgen treated GSFs reveal transcriptional programs involved in development and differentiation and are therefore an important tool to study male genital outgrowth pre- and postnatally. Interestingly, these transcriptional programs include neuronal, renal, muscle and cardiovascular development, where the AR has been shown to be involved [42–46]. Several AR-target genes identified here are also involved in prostate cancer progression. This study therefore helps towards a better understanding of androgen-dependent processes in genital as well as non-genital tissues.

## Conclusion

This study shows that genital skin fibroblasts, despite being terminally differentiated, retain androgen responsiveness and reveal transcriptional programs relevant to male genital outgrowth. By identifying robust AR-regulated genes, including *APOD*, *FKBP5*, *AOX1*, *HGF*, and *HAND2*, our findings highlight known and novel candidates that may contribute to the molecular basis of male sex differentiation. Such work is particularly important for understanding the pathogenesis of 46,XY DSDs, where incomplete androgen signalling leads to diverse phenotypes. Expanding the catalogue of androgen-regulated genes in human tissues not only provides valuable diagnostic targets but also deepens our insight into the complex regulatory networks underlying androgen action in development and disease.

## Materials and methods

### Patient material

Male control scrotum-derived genital fibroblasts (GSF-S) were obtained from fertile adult patients with typical external genitalia virilization who underwent vasectomy (*n* = 2) and from patients under the age of 18 who underwent orchidopexy due to maldescended testes (*n* = 4) but with typical external genitalia, i.e., no hypospadias. In addition, we used male control foreskin fibroblasts (GSF-F) from patients who underwent circumcision for cultural reasons or phimosis (*n* = 6) [5]. We also included GSFs from Labia majora (GSF-L.Maj) (*n* = 2) and Labia minora (GSF-L.Min) (*n* = 2) samples of CAIS individuals carrying loss-of-function mutations in the *AR*. The GSFs used and a visualisation of CAIS mutations can be seen in Supplementary file 1: Table S1 and Fig. S1.

### Cell culture and hormone induction

GSFs were cultured in phenol red free Dulbecco’s modified Eagle’s medium (DMEM) supplemented with 10% fetal bovine serum (FBS, MaxSpec), 100 units/ml penicillin/streptomycin, 2 mM L-glutamine and 20 mM HEPES buffer (all purchased from Life Technologies, Carlsbad, USA) and incubated at 37 °C in a 5% CO2 incubator (Binder GmbH Tuttlingen, Germany). For hormone induction, cells were seeded in four 10 cm dishes: two dishes with a concentration of 2.8 × 10^5^ cells for 72 h hormone treatment and two dishes with a concentration of 3.5 × 10^5^ cells for 48 h hormone treatment. After 24 h, cells were washed three times with PBS (Life Technologies, Carlsbad, USA), and medium containing 0.1% charcoal-treated FBS was added to the cells. DHT (Sigma-Aldrich, St. Louis, USA), dissolved in ethanol (EtOH) (JT Baker, Phillipsburg, USA) was added once to two dishes at a final concentration of 10 nM, while the control dishes were treated with the same volume of ethanol. The concentration of 10nM DHT was chosen based on serial dilution experiments in order to reach maximal activation levels of the AR [5]. Cells were incubated for 48 h and 72 h at 37 °C with 5% CO2, after which they were lysed in RNA-extraction buffer (RLT; Qiagen, Hilden, Germany). Hence, each GSF line underwent four different experimental conditions: EtOH and DHT treatments at two time points (48 h and 72 h). The use of 48 h and 72 h treatment was based on previous experiments showing a robust *APOD* induction after 72 h treatment [5]. We did not include a 24 h hormone treatment as we observed an *APOD* induction below two-fold in a trial experiment (Supplementary file 1: Fig. S6; Supplementary file 5).

### RNA extraction

Total RNA was extracted using the RNeasy Mini Kit (Qiagen, Hilden, Germany), including on-column DNase digestion (RNAse-Free DNase Set, Qiagen, Hilden, Germany) to eliminate residual DNA, following the manufacturer’s protocol. RNA quantity and quality were measured using a NanoDrop ND-1000 Spectrophotometer (Peqlab, Erlangen, Germany) and an Agilent 2100 Bioanalyzer using the RNA 6000 Nano Chip Kit (Agilent, Santa Clara, USA), respectively. RNA purity was assessed using A260/A280 ratios, with values above 2.0 indicating pure RNA without protein contamination. All samples showed ratios above 2.0, consistent with high purity. RNA integrity was assessed using RNA Integrity Numbers (RINs) obtained from the Agilent 2100 Bioanalyzer. The RIN algorithm evaluates the ratio and distribution of ribosomal RNA peaks (28 S and 18 S) and overall RNA degradation, providing a quality score from 1 (completely degraded) to 10 (highly intact).

### Library preparation and RNA sequencing

All RNA samples processed for mRNA sequencing had an RNA Integrity Number (RIN) score ≥ 8, as determined by the Agilent 2100 Bioanalyzer (Agilent, Santa Clara, USA), indicating high-quality and intact RNA suitable for transcriptome analysis. Total RNA (1 µg) was converted into sequencing libraries using the Illumina Stranded mRNA Prep Ligation kit (Illumina, Cat. No. 20040532, San Diego, USA). Sample-specific barcoding was achieved utilizing Illumina Unique Dual (UD) Set A index adapters, employing distinct dual indexes (I7-10 and I5-10) (Illumina, Cat. No. 20091655; San Diego, USA). The quality of the amplified cDNA was validated with the Bioanalyzer 2100 using the High Sensitivity DNA kit (Agilent, Santa Clara, USA), and the concentration was measured using the Qubit DNA HS Assay (Invitrogen, Carlsbad, USA). High-quality DNA libraries were pooled equimolarly and a 650pM pool was sequenced using paired-end 200 bp reads on the NextSeq 2000 system (Illumina, San Diego, USA) with NextSeq 2000 P3 Reagents (200 cycles; Illumina, Cat. No. 20040560, San Diego, USA). Sequencing data conversion and demultiplexing were performed using bcl2fastq2 v2.20 (Illumina). Gene-level count matrices were generated from the FASTQ files using the community-driven nf-core/rnaseq pipeline v3.12.0 [47]. The pipeline included adapter trimming (TrimGalore v0.6.7), contamination removal (SortMeRNA v4.3.4 and BBMap-BBSplit v39.01), alignment (STAR 2.7.10a) to hg38, transcript-level quantification (Salmon v1.10.1), and comprehensive quality control (FastQC v0.11.9, RESeQC v3.0.1, dupRadar v1.28.0, and MultiQC v1.14). Transcript-level counts were converted to gene-level counts using tximeta-tximport v1.12.0. As the samples were processed in two separate sequencing runs (one for male control and one for CAIS samples), batch correction was applied to account for run-specific effects. The datasets were combined and processed together using Salmon (which includes STAR aligner support), ensuring consistent quantification across both batches.

### Differential gene expression analysis

Transcriptomic analysis was performed using Shiny-Seq [48], an RNA-sequencing analysis pipeline based on DESeq2. The analysis utilised a count matrix (summarising gene expression levels across samples) and a metadata table (describing sample attributes) as input data to compare gene expression levels between different experimental conditions. The dataset was normalized using the DESeq2 package with a filtering cut-off of 10 raw reads. For hierarchical clustering samples were classified based on a combination of tissue type, treatment type and incubation time for male control and CAIS. The composite condition “Tissue_Treatment_Time” was chosen for normalization to account for variation across these factors. To address batch effects, Surrogate Variable Analysis (SVA) [49] was applied, estimating ‘10’ surrogate variables for male control samples and ‘2’ for CAIS samples. For tissue-specific gene expression analysis, ‘5’ surrogate variables were estimated for scrotum/foreskin derived samples, and ‘1’ for labia majora/minora-derived samples.

Differentially expressed genes in each tissue were identified by comparing DHT-treated versus ethanol-treated cells at 48 h and 72 h time points. Genes with log2 fold change (log2FC) >0 were considered up-regulated, and those with log2FC < 0 were considered down-regulated, with a significance threshold of adjusted p-value (padj) ≤ 0.05. For a more stringent analysis, we selected DEGs with log2FC ≥ 0.5 or log2FC ≤ −0.5 and a padj ≤ 0.05. The list of DEGs is provided in Supplementary file 2. The distribution of DEGs was visualised using the MaGIC Volcano Plot Tool [50], which plots the magnitude of change (log2FC) against statistical significance (-log10 padj). Venn diagrams were created using Venny 2.1.0 [51].

### Quantitative Real-Time PCR (qRT-PCR)

Total RNA (500ng) was reverse transcribed using QuantiTect Reverse Transcription Kit (Qiagen, Hilden, Germany). qRT-PCR was carried out using the QuantiTect SYBR Green master mix (Qiagen, Hilden, Germany) with gene-specific primers, tested in duplicate for each sample. For hormone induction trial experiments at 24 h and 48 h *APOD* mRNA expression was measured (Supplementary file 1: Fig. S6; Supplementary file 5). For the validation of the RNA-Seq results, six up-regulated genes (*MYOCD*, *FKBP5*, *HSD11B1*, *FAM105A*, *FAM107A*, *STEAP4*) were selected for analysis. qRT-PCR was performed on samples from four male control individuals (GSF-F, *n* = 2; GSF-S, *n* = 2) and two CAIS individuals (L.Maj, *n* = 1; L.Min, *n* = 1), with both DHT- and EtOH-treated samples collected at 72 h. The housekeeping gene succinate dehydrogenase complex, subunit A (*SDHA*), was used to normalise the gene expression data (Supplementary file 3). Primers for *APOD*,* SDHA*,* MYOCD*, *FKBP5*, and *HSD11B1* were obtained from Qiagen (Hilden, Germany) and used according to the manufacturer’s instructions, while primers for *FAM105A*, *FAM107A* and *STEAP4* were designed using Primer3 (Supplementary file 1: Table S3) and purchased from Biomers GmbH (Ulm, Germany). Thermal cycling conditions were as follows: initial denaturation at 95 °C for 10 min, followed by annealing at 55 °C (MYOCD, FKBP5, HSD11B1, FAM105A, FAM107A) or 58 °C (STEAP4) for 30 s and elongation at 72 °C for 30 s for a total of 45 cycles. qRT-PCR was performed on a LightCycler 480 (Roche, Basel, Switzerland) with SYBR Green (Qiagen, Hilden, Germany) detection. For each sample, Ct values from technical duplicates were averaged. The relative expression of mRNA was calculated using the 2^−ΔΔCt method, where ΔCt = Ct_target – Ct_housekeeping gene (SDHA). A melting curve analysis was performed at the end of each run to confirm amplicon specificity.

### ARE motif scanning

Chromatin-immunoprecipitation-sequencing (ChIP-seq) data from GEO sources: GSM3148986 and GSM3148988 for ethanol and androgen treated LNCaP cells [17] and GSM1410768 and GSM1410785 for ethanol and androgen treated VCaP cells [18] were visualized using the Cistrome data browser and checked for androgen-induced AR-binding within the gene region including 25 kb upstream of the transcriptional start site. The binding area was then screened for the canonical AR binding site motif (AGAACANNNTGTTCT) using the Find Individual Motif Occurrences (FIMO) software from MEME suite 5.5.7.

### Gene ontology analysis

Gene Ontology enrichment analysis was performed using PANTHER [52] with significantly up- and down-regulated DEGs, as well as those with log2FC ≥ 0.5 or ≤, applying a false discovery rate (FDR) threshold of 0.05. The resulting data was visualized using R Studio (R version 4.4.0 (ucrt) [53] to generate bar plots. Biological processes were sorted by fold enrichment from highest to lowest, and the top 20 terms were included in the plot. For genes with log2FC ≥ 0.5 or ≤, the top 10 enriched pathways were plotted. The list of significantly enriched GO biological process terms is provided in Supplementary file 4.

## Supplementary Information

Supplementary Material 1.

Supplementary Material 2.

Supplementary Material 3.

Supplementary Material 4.

Supplementary Material 5.

Supplementary Material 6.

## Data Availability

Sequence data that support the findings of this study have been deposited to the Gene Expression Omnibus (https://www.ncbi.nlm.nih.gov/geo/query/acc.cgi?acc=GSE300211).

## References

[1] Brinkmann AO, Klaasen P, Kuiper GGJM, van der Korput JAGM, Bolt J, de Boer W, et al. Structure and function of the androgen receptor. Urol Res. 1989;17(2):87–93. 2734982 10.1007/BF00262026

[2] Mongan NP, Tadokoro-Cuccaro R, Bunch T, Hughes IA. Androgen insensitivity syndrome. Best Pract Res Clin Endocrinol Metab. 2015;29(4):569–80. 26303084 10.1016/j.beem.2015.04.005

[3] Hornig NC, Holterhus PM. Molecular basis of androgen insensitivity syndromes. Mol Cell Endocrinol. 2021;523:111146. 33385475 10.1016/j.mce.2020.111146

[4] Ahmed SF, Bashamboo A, Lucas-Herald A, McElreavey K. Understanding the genetic aetiology in patients with XY DSD. Br Med Bull. 2013;106(1):67–89. 23529942 10.1093/bmb/ldt008

[5] Hornig NC, Ukat M, Schweikert HU, Hiort O, Werner R, Drop SLS, et al. Identification of an *AR* mutation-negative class of androgen insensitivity by determining endogenous AR activity. J Clin Endocrinol Metab. 2016;101(11):4468–77. 27583472 10.1210/jc.2016-1990 PMC5095254

[6] Coffey K, Robson CN. Regulation of the androgen receptor by post-translational modifications. J Endocrinol. 2012;215(2):221–37. 22872761 10.1530/JOE-12-0238

[7] Heemers HV, Tindall DJ. Androgen receptor (AR) coregulators: a diversity of functions converging on and regulating the AR transcriptional complex. Endocr Rev. 2007;28(7):778–808. 17940184 10.1210/er.2007-0019

[8] van der Steen T, Tindall DJ, Huang H. Posttranslational modification of the androgen receptor in prostate cancer. Int J Mol Sci. 2013;14(7):14833–59. 23863692 10.3390/ijms140714833 PMC3742275

[9] Grötsch H, Kunert M, Mooslehner KA, Gao Z, Struve D, Hughes IA, et al. RWDD1 interacts with the ligand binding domain of the androgen receptor and acts as a coactivator of androgen-dependent transactivation. Mol Cell Endocrinol. 2012;358(1):53–62. 22406838 10.1016/j.mce.2012.02.020

[10] Knerr J, Werner R, Schwan C, Wang H, Gebhardt P, Grötsch H, et al. Formin-mediated nuclear actin at androgen receptors promotes transcription. Nature. 2023;617(7961):616–22. 36972684 10.1038/s41586-023-05981-1

[11] Holterhus PM, Hiort O, Demeter J, Brown PO, Brooks JD. Differential gene-expression patterns in genital fibroblasts of normal males and 46,XY females with androgen insensitivity syndrome: evidence for early programming involving the androgen receptor. Genome Biol. 2003;4(6):R37. 12801411 10.1186/gb-2003-4-6-r37 PMC193616

[12] Holterhus PM, Deppe U, Werner R, Richter-Unruh A, Bebermeier JH, Wünsch L, et al. Intrinsic androgen-dependent gene expression patterns revealed by comparison of genital fibroblasts from normal males and individuals with complete and partial androgen insensitivity syndrome. BMC Genomics. 2007;8:376. 17945006 10.1186/1471-2164-8-376 PMC2212662

[13] Holterhus PM, Bebermeier JH, Werner R, Demeter J, Richter-Unruh A, Cario G, et al. Disorders of sex development expose transcriptional autonomy of genetic sex and androgen-programmed hormonal sex in human blood leukocytes. BMC Genomics. 2009;10:292. 19570224 10.1186/1471-2164-10-292 PMC2713997

[14] Rodie ME, Mudaliar MAV, Herzyk P, McMillan M, Boroujerdi M, Chudleigh S, et al. Androgen-responsive non-coding small RNAs extend the potential of HCG stimulation to act as a bioassay of androgen sufficiency. Eur J Endocrinol. 2017;177(4):339–46. 28733293 10.1530/EJE-17-0404

[15] Appari M, Werner R, Wünsch L, Cario G, Demeter J, Hiort O, et al. Apolipoprotein D (APOD) is a putative biomarker of androgen receptor function in androgen insensitivity syndrome. J Mol Med. 2009;87(6):623–32. 19330472 10.1007/s00109-009-0462-3 PMC5518750

[16] Tanase-Nakao K, Mizuno K, Hayashi Y, Kojima Y, Hara M, Matsumoto K, et al. Dihydrotestosterone induces minor transcriptional alterations in genital skin fibroblasts of children with and without androgen insensitivity. Endocr J. 2019;66(4):387–93. 30787207 10.1507/endocrj.EJ18-0494

[17] Tran MGB, Bibby BAS, Yang L, Lo F, Warren AY, Shukla D, et al. Independence of HIF1a and androgen signaling pathways in prostate cancer. BMC Cancer. 2020;20(1):469. 32450824 10.1186/s12885-020-06890-6 PMC7249645

[18] Takayama Kichi, Suzuki T, Fujimura T, Urano T, Takahashi S, Homma Y, et al. CtBP2 modulates the androgen receptor to promote prostate cancer progression. Cancer Res. 2014;74(22):6542–53. 25228652 10.1158/0008-5472.CAN-14-1030

[19] Amato CM, Yao HHC. Developmental and sexual dimorphic atlas of the prenatal mouse external genitalia at the single-cell level. Proc Natl Acad Sci. 2021;118(25):e2103856118. 34155146 10.1073/pnas.2103856118 PMC8237666

[20] Rassart E, Bedirian A, Do Carmo S, Guinard O, Sirois J, Terrisse L, et al. Apolipoprotein D. Biochim Biophys Acta. 2000;1482(1–2):185–98. 11058760 10.1016/s0167-4838(00)00162-x

[21] Fyfe-Desmarais G, Desmarais F, Rassart É, Mounier C. Apolipoprotein D in oxidative stress and inflammation. Antioxidants. 2023. 10.3390/antiox12051027. 37237893 10.3390/antiox12051027 PMC10215970

[22] Huang M, Zheng J, Chen L, You S, Huang H. Role of apolipoproteins in the pathogenesis of obesity. Clin Chim Acta. 2023;545:117359. 37086940 10.1016/j.cca.2023.117359

[23] Zeng C, Spielman AI, Vowels BR, Leyden JJ, Biemann K, Preti G. A human axillary odorant is carried by Apolipoprotein D. Proc Natl Acad Sci U S A. 1996;93(13):6626–30. 8692868 10.1073/pnas.93.13.6626 PMC39076

[24] Ni L, Yang CS, Gioeli D, Frierson H, Toft DO, Paschal BM. FKBP51 promotes assembly of the Hsp90 chaperone complex and regulates androgen receptor signaling in prostate cancer cells. Mol Cell Biol. 2010;30(5):1243–53. 20048054 10.1128/MCB.01891-08 PMC2820886

[25] Maeda K, Habara M, Kawaguchi M, Matsumoto H, Hanaki S, Masaki T, et al. FKBP51 and FKBP52 regulate androgen receptor dimerization and proliferation in prostate cancer cells. Mol Oncol. 2022;16(4):940–56. 34057812 10.1002/1878-0261.13030 PMC8847985

[26] Magee JA, Chang L, wei, Stormo GD, Milbrandt J, Direct. Androgen Receptor-Mediated regulation of the FKBP5 gene via a distal enhancer element. Endocrinol [Internet]. 2006;147(1):590–8. 10.1210/en.2005-1001 16210365

[27] Wei J, Yin L, Li J, Wang J, Pu T, Duan P, et al. Bidirectional cross-talk between MAOA and AR promotes hormone-dependent and castration-resistant prostate cancer. Cancer Res. 2021;81(16):4275–89. 34167949 10.1158/0008-5472.CAN-21-0198 PMC8373824

[28] Ceccarelli DF, Ivantsiv S, Mullin AA, Coyaud E, Manczyk N, Maisonneuve P, et al. FAM105A/OTULINL is a pseudodeubiquitinase of the OTU-Class that localizes to the ER membrane. Structure. 2019;27(6):1000–e10126. 31056421 10.1016/j.str.2019.03.022 PMC6551266

[29] Quartier A, Chatrousse L, Redin C, Keime C, Haumesser N, Maglott-Roth A, et al. Genes and pathways regulated by androgens in human neural cells, potential candidates for the male excess in autism spectrum disorder. Biol Psychiatry. 2018;84(4):239–52. 29428674 10.1016/j.biopsych.2018.01.002

[30] Nakajima H, Koizumi K. Family with sequence similarity 107: a family of stress responsive small proteins with diverse functions in cancer and the nervous system (Review). Biomed Rep. 2014;2(3):321–5. 24748967 10.3892/br.2014.243 PMC3990222

[31] Huff SD, Chaykin S. Genetic and androgenic control of N1-methylnicotinamide oxidase activity in mice. J Biol Chem. 1967;242(6):1265–70. 4225773

[32] Stanulović M, Chaykin S. Aldehyde oxidase: catalysis of the oxidation of N 1 -methylnicotinamide and pyridoxal. Arch Biochem Biophys. 1971;145(1):27–34. 4256441 10.1016/0003-9861(71)90005-1

[33] Huff SD, Chaykin S. Kinetics of testosterone action, in vivo, on liver N-methylnicotinamide oxidase activity in mice. Endocrinology. 1968;83(6):1259–67. 4235399 10.1210/endo-83-6-1259

[34] Pace AJ, Lee E, Athirakul K, Coffman TM, O’Brien DA, Koller BH. Failure of spermatogenesis in mouse lines deficient in the Na(+)-K(+)-2Cl(-) cotransporter. J Clin Invest. 2000;105(4):441–50. 10683373 10.1172/JCI8553 PMC289162

[35] Ricci G, Catizone A. Pleiotropic activities of HGF/c-Met system in testicular physiology: paracrine and endocrine implications. Front Endocrinol (Lausanne). 2014;5:38. 24772104 10.3389/fendo.2014.00038 PMC3982073

[36] Ferguson CA, Firulli BA, Zoia M, Osterwalder M, Firulli AB. Identification and characterization of Hand2 upstream genomic enhancers active in developing stomach and limbs. Dev Dyn. 2024;253(2):215–32. 37551791 10.1002/dvdy.646 PMC11365009

[37] Seifert AW, Bouldin CM, Choi KS, Harfe BD, Cohn MJ. Multiphasic and tissue-specific roles of Sonic Hedgehog in cloacal septation and external genitalia development. Development. 2009;136(23):3949–57. 19906862 10.1242/dev.042291 PMC2778742

[38] Perriton CL, Powles N, Chiang C, Maconochie MK, Cohn MJ. Sonic hedgehog signaling from the urethral epithelium controls external genital development. Dev Biol. 2002;247(1):26–46. 12074550 10.1006/dbio.2002.0668

[39] O’Shaughnessy KL, Dahn RD, Cohn MJ. Molecular development of Chondrichthyan claspers and the evolution of copulatory organs. Nat Commun. 2015;6:6698. 25868783 10.1038/ncomms7698 PMC4403318

[40] Cunha GR, Vezina CM, Isaacson D, Ricke WA, Timms BG, Cao M, et al. Development of the human prostate. Differentiation. 2018;103:24–45. 30224091 10.1016/j.diff.2018.08.005 PMC6234090

[41] Marques P, De Sousa Lages A, Skorupskaite K, Rozario KS, Anderson RA, George JT. Physiology of GnRH and Gonadotrophin Secretion. 2000.

[42] Ward PJ, Davey RA, Zajac JD, English AW. Neuronal androgen receptor is required for activity dependent enhancement of peripheral nerve regeneration. Dev Neurobiol. 2021;81(4):411–23. 33864349 10.1002/dneu.22826 PMC8291079

[43] Rizk J, Sahu R, Duteil D. An overview on androgen-mediated actions in skeletal muscle and adipose tissue. Steroids. 2023;199:109306. 37634653 10.1016/j.steroids.2023.109306

[44] Huang CK, Lee SO, Chang E, Pang H, Chang C. Androgen receptor (AR) in cardiovascular diseases. J Endocrinol. 2016;229(1):R1-16. 26769913 10.1530/JOE-15-0518 PMC4932893

[45] Lucas-Herald AK, Touyz RM. Androgens and androgen receptors as determinants of vascular sex differences across the lifespan. Can J Cardiol. 2022;38(12):1854–64. 36156286 10.1016/j.cjca.2022.09.018

[46] Xiong L, Liu J, Han SY, Koppitch K, Guo JJ, Rommelfanger M, et al. Direct androgen receptor control of sexually dimorphic gene expression in the mammalian kidney. Dev Cell. 2023;58(21):2338–e23585. 37673062 10.1016/j.devcel.2023.08.010 PMC10873092

[47] Patel H, Ewels P, Manning J, Garcia MU, Peltzer A, Hammarén R, et al. nf-core/rnaseq: nf-core/rnaseq v3.18.0 -. Lithium Lynx [Internet]. Zenodo; 2024.

[48] Sundararajan Z, Knoll R, Hombach P, Becker M, Schultze JL, Ulas T. Shiny-seq: advanced guided transcriptome analysis. BMC Res Notes. 2019;12(1):432. 31319888 10.1186/s13104-019-4471-1 PMC6637470

[49] Leek JT, Johnson WE, Parker HS, Jaffe AE, Storey JD. The < tt > sva package for removing batch effects and other unwanted variation in high-throughput experiments. Bioinformatics. 2012;28(6):882–3. 22257669 10.1093/bioinformatics/bts034 PMC3307112

[50] Lemenze A. MaGIC-Analytics/magic-volcanoes: V1 release [Internet]. Zenodo; 2024.

[51] Oliveros JC. Venny: an interactive tool for comparing lists with Venn diagrams. CNB-CSIC: BioinfoGP; 2007.

[52] Mi H, Muruganujan A, Casagrande JT, Thomas PD. Large-scale gene function analysis with the PANTHER classification system. Nat Protoc. 2013;8(8):1551–66. 23868073 10.1038/nprot.2013.092 PMC6519453

[53] R Core Team. R: A Language and Environment for Statistical Computing. Vienna, Austria; 2024.

