# Supplementary material for: Comprehensive androgen-dependent transcriptome analysis in human genital tissue
Source: BMC Genomics. 2025 Nov 17;26:1047. doi: 10.1186/s12864-025-12212-6 (PMC12621392; doi:10.1186/s12864-025-12212-6)
Supplement: Supplementary file 1 — Supplementary Material 1. [file 12864_2025_12212_MOESM1_ESM.docx]

| **Sample ID** | **Group** | **Genetic variants** | **Age (years) at biopsy** | **Tissue Type - Origin** |
| --- | --- | --- | --- | --- |
| GSF-35 | Male control/  GSF-S |  | 0.88 | Scrotum |
| GSF-41 |  |  | 0.99 |  |
| GSF-170 |  |  | 10.45 |  |
| GSF-33 |  |  | 10.40 |  |
| GSF-3 |  |  | 30.93 |  |
| GSF-28 |  |  | 28.85 |  |
| GSF-123 | Male control/  GSF-F |  | 6.78 | Foreskin |
| GSF-122 |  |  | 7.67 |  |
| GSF-121 |  |  | 8.76 |  |
| GSF-119 |  |  | 10.45 |  |
| GSF-138 |  |  | 21.07 |  |
| GSF-139 |  |  | 44.18 |  |
| GSF-154 | CAIS/  GSF-L. Min | ChrX:66931475 A>G; p.(Asn706Ser) | 41 | Labia minora |
| GSF-156 |  | ChrX:66941708-66941709del; p.(Val786Profs*42) | 17 |  |
| GSF-73 | CAIS/  GSF-L. Maj | ChrX:66931532 G>T (spice site mutation) | 17,2 | Labia majora |
| GSF-76 |  | ChrX:66942760-66942761insA; p.(Asn849Lysfs*31) | 2,64 |  |

**Table S1** List of GSFs used

**Fig. S1** Visualisation of CAIS mutations in Integrative Genomics Viewer (IGV) using the hg19 reference genome. **A)** chrX:66931532 G>T splice-site mutation in sample GF-73. **B)** chrX:66942760-66942761insA variant in sample GF-76, resulting in the p.(Asn849Lysfs*31) frameshift mutation.

**C)** chrX:66931475 A>G variant in sample GF-154, causing the p.(Asn706Ser) amino acid substitution. **D)** chrX:66941708-66941709del variant in sample GF-156, leading to the p.(Val786Profs*42) frameshift mutation.

**
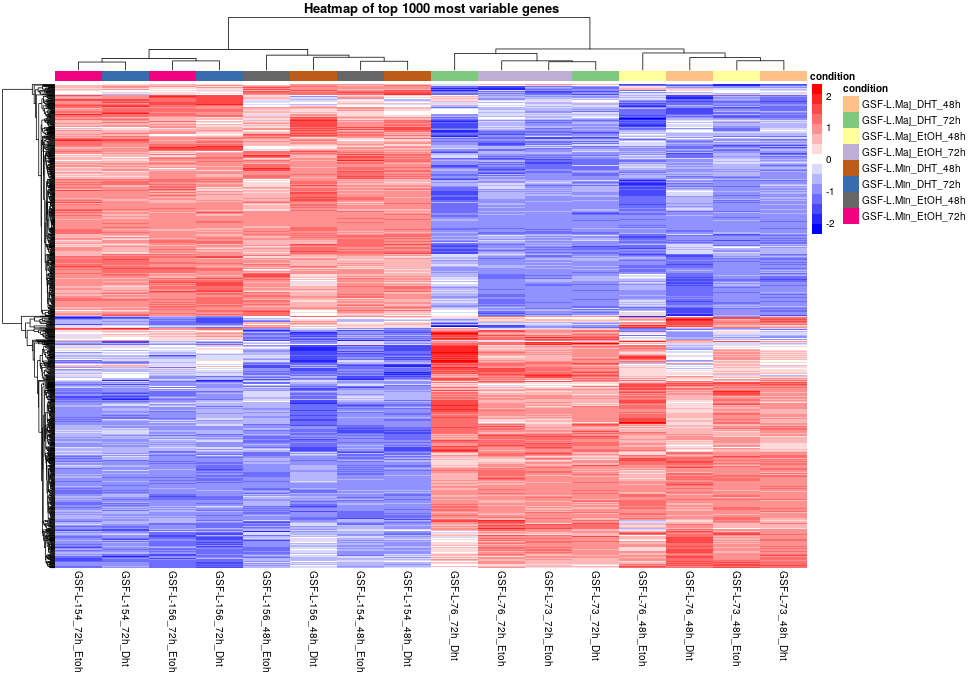
**

**Fig. S2** Heatmap displaying the expression profiles of the top 1,000 most variable genes across all conditions in CAIS samples. Each column represents an individual sample, grouped by tissue type (L.Maj or L.Min), treatment (DHT or EtOH), and time point (48 h or 72 h), as indicated by the color-coded bar above. Each row corresponds to a gene. The colour scale reflects gene expression levels, with red representing high expression and blue indicating low expression. Hierarchical clustering was applied to both genes and samples to visualize expression similarities and condition-specific patterns.

**Fig. S3** Venn diagram showing the number and percentage of DEGs uniquely or commonly regulated at both time points 48 h and 72 h. Up-regulated genes: **A)** GSF-F and **B)** GSF-S. Down-regulated genes: **C)** GSF-F and **D)** GSF-S.

**Fig. S4:** Venn diagram showing the number and percentage of DEGs uniquely or commonly regulated at 48 h and 72 h time points. Up-regulated genes with log2FC ≥ 0.5: **A)** GSF-F and **B)** GSF-S. Down-regulated genes with log2FC ≤ -0.5: **C)** GSF-F and **D)** GSF-S.

**A**

**B**

**Fig. S5:** Validation of the RNA-Seq using qRT-PCR. Relative mRNA expression between ethanol- and DHT-treatment of six selected genes was assessed in control samples (GSF-28: , GSF-35: , GSF-119: , GSF-139: ) **(A).**  DHT-induced target gene expression (shown as ratio DHT/EtOH) of the genes *MYOCD*, *FKBP5*, *HSD11B1* and *FAM105A* in control samples as in (A) and CAIS samples (GSF-73, GSF-156) **(B)**. Gene expression was normalized to the housekeeping gene *SDHA*.

| **Up-regulated genes (GSF-F and GSF-S at 48h and 72h)** | **AR ChIPseq peak in LnCaP (distance to TSS in bp)** | **AR ChIPseq peak in VCaP (distance to TSS in bp)** | **Canonical AR binding site motif: AGAACANNNTGTTCT** | **p-value/ q-value** |
| --- | --- | --- | --- | --- |
| *AOX1* | +80015 | +80015 | AGAACAATCTGTTAG | 4.01e-05 / 0.0104 |
| *APOD* | -485 | -485 | GGAACATGGAGTTCC | 8.62e-05 / 0.0464 |
| *ASPN* | no | no |  |  |
| *CCDC68* | +1927 | +1927 | AGAACACAGTGTCCT | 8.35e-07 / 0.000231 |
| *CD82* | -932 | -932 | AGCACTGGTTGTTCT | 9.95e-06 / 0.0232 |
| *CERS6* | +71232 | +71232 | AGAACACTCTGTGCT | 8.35e-07 / 0.001 |
| *CILP* | no | no |  |  |
| *ENPP1* | no | no |  |  |
| *ERCC6* | +34037 | +34037 | AGAGCATGCTGTTTT | 2.69e-05 / 0.0248 |
| *FAM105A* | +7808 | +7808 | AGGACACCGTGTGCT | 4.49e-06 / 0.00344 |
| *FAM107A* | +205 | +205 | GGAACATCATGTCCA | 0.000142 / 0.046 |
| *FKBP5* | +1682 | +1682 | GGAACACGAGGTTCT | 9.95e-06 / 0.00476 |
| *IMPA2* | -4365,  -4931 | -4365,  -4931 | AGAAAAAGCTGATTT,  TGGCCAGGCTGGTCT | 0.000376 / 0.0899, 0.000524 / 0.089 |
| *KIF26B* | +24860 | +24860 | AGAACATCCTGTCCA | 9.95e-06/ 0.00813 |
| *LINC00968* | no | no |  |  |
| *MAOA* | no | no |  |  |
| *MYOCD* | +15790 | +15790 | AGAACAGTGTGTACC | 9.95e-06 / 0.00889 |
| *NR2F1* | no | no |  |  |
| *OMD* | no | no |  |  |
| *RGCC* | no | no |  |  |
| *SLC38A11* | no | no |  |  |
| *SORBS1* | no | no |  |  |
| *TCN2* | no | no |  |  |

| **Down-regulated genes (GSF-F and GSF-S at 48h and 72h)** | **AR ChIPseq peak in LnCAP (distance to TSS in bp)** | **AR ChIPseq peak in VCaP (distance to TSS in bp)** | **Canonical AR binding site motif: AGAACANNNTGTTCT** | **p-value/ q-value** |
| --- | --- | --- | --- | --- |
| *CD36* | no | no |  |  |
| *L1CAM* | no | no |  |  |
| *PRELP* | no | no |  |  |

**Table S2** DHT-induced AR-binding in LnCaP and VCaP in commonly up- or down-regulated genes at both time points in both tissues with a minimum log2FC of ±0.5. Found AR-binding motifs based on the canonical AR-binding motif are shown with the corresponding p- and q-values.

TSS = transcriptional start site.

**Fig. S6** APOD induction over time. Male control GSF-28 were induced with 10nM DHT or treated with ethanol for 24 h and 48 h. The mean DHT induction after 24 h was 1.8, the induction after 48h was 2.5 (see Supplementary file 5)

| **Target gene** | **Forward Primer** | **Reverse Primer** | **Annealing Temperature (ͦ c)** |
| --- | --- | --- | --- |
| *FAM105A* | 5’-CGCAGGAAGTGACCAAGTT-3’ | 5’-GCTTGTGCCCTGAATATAAATGT-3’ | 55 |
| *FAM107A* | 5’-ATCAAGAAGAAGAAGGAG-3’ | 5’-TTCCCTGACTTTAATAAAC-3’ | 55 |
| *STEAP 4* | 5’-CCGTTACCCAGGCAATACTC-3’ | 5’-TCTCCAGTTGACTGCATTGC-3’ | 58 |

**Table S3** Oligonucleotide primers used for RT-qPCR ampliﬁcation of target genes.
